# Eplerenone-Resistant Salt-Sensitive Hypertension in Nedd4-2 C2 KO Mice

**DOI:** 10.3390/ijms18061250

**Published:** 2017-06-11

**Authors:** Tabito Kino, Tomoaki Ishigami, Tsumugi Murata, Hiroshi Doi, Rie Nakashima-Sasaki, Lin Chen, Michiko Sugiyama, Kengo Azushima, Hiromichi Wakui, Shintaro Minegishi, Kouichi Tamura

**Affiliations:** 1 Department of Medical Science and Cardiorenal Medicine, Graduate School of Medicine, Yokohama City University, 3-9 Fukuura, Kanazawa-ku, Yokohama 236-0004, Japan; kino-tabito@umin.ac.jp (T.K.); e123080g@yokohama-cu.ac.jp (T.M.); t156052b@yokohama-cu.ac.jp (H.D.); musika0720@yahoo.co.jp (R.N.-S.); mysterylin@foxmail.com (L.C.); vn_nv2525smile@yahoo.co.jp (M.S.); azushima@yokohama-cu.ac.jp (K.A.); hiro1234@yokohama-cu.ac.jp (H.W.); shintaro.minegish@gmail.com (S.M.); tamukou@yokohama-cu.ac.jp (K.T.); 2 Cardiovascular and Metabolic Disorders Program, Duke-NUS Medical School, 8 College Road, Singapore 169857, Singapore

**Keywords:** salt sensitivity, hypertension, Nedd4-2 C2, eplerenone, amiloride, resistant hypertension

## Abstract

The epithelial sodium channel (ENaC) plays critical roles in maintaining fluid and electrolyte homeostasis and is located in the aldosterone-sensitive distal nephron (ASDN). We previously found that Nedd4-2 C2 knockout (KO) mice showed salt-sensitive hypertension with paradoxically enhanced *ENaC* gene expression in ASDN under high oral salt intake. Eplerenone (EPL), a selective aldosterone blocker, is a promising therapeutic option for resistant or/and salt-sensitive hypertension. We examined the effect of EPL on Nedd4-2 C2 KO mice with respect to blood pressure, metabolic parameters, and molecular level changes in ASDN under high oral salt intake. We found that EPL failed to reduce blood pressure in KO mice with high oral salt intake and upregulated *ENaC* expression in ASDN. Thus, salt-sensitive hypertension in Nedd4-2 C2 KO was EPL-resistant. Gene expression analyses of laser-captured specimens in ASDN suggested the presence of non-aldosterone-dependent activation of *ENaC* transcription in ASDN of Nedd4-2 C2 KO mice, which was abolished by amiloride treatment. Our results from Nedd4-2 C2 KO mice suggest that enhanced *ENaC* gene expression is critically involved in salt-sensitive hypertension under certain conditions of specific enzyme isoforms for their ubiquitination.

## 1. Introduction

Salt sensitivity and hypertension are major human health problems because cardiovascular morbidity and mortality due to hypertension are increasing worldwide [1,2,3]. Recent clinical practice guidelines for managing hypertension in various countries indicate that appropriate blood pressure lowering is necessary for reducing cardiovascular mortality [4,5,6,7]. “The lower, the better” strategies for blood pressure management have been validated by cumulative clinical trials [8] and subsequent systematic reviews [9]. The main pharmacological therapeutic targets for hypertension include the renin-angiotensin system, sodium channels in renal tubules, and increased vascular tone in peripheral arteries, which are treatable by angiotensin-converting enzyme inhibitors, angiotensin type I receptor blockers, diuretics (thiazide derivatives), and calcium channel blockade, respectively. Among subjects with hypertension, some failed to show adequate improvement despite appropriate prescription of the abovementioned basic representative anti-hypertensive drugs, a condition known as resistant hypertension [10,11]. Therefore, treatments for resistant hypertension are currently being studied as therapeutic options in clinical hypertension [12]. High oral salt intake in rural northern Japan and a higher prevalence of hypertensive disease have been reported in previous hypertension studies [13,14], and subsequent global epidemiological studies have supported this relationship [15]. Excessive oral salt intake in subjects with salt sensitivity results in hypertension and is thought to be involved in the pathophysiological basis of resistant hypertension [16]. Eplerenone and spironolactone are therapeutic options for subjects with resistant hypertension, suggesting that residual activation of aldosterone signaling in distal tubules is the pathophysiological basis of this condition [17,18,19,20]. Recently, we used Nedd4-2 C2 knock out (KO) mice to demonstrate that loss of the Nedd4-2 C2 isoform causes salt-sensitive hypertension under conditions of high dietary salt intake in vivo [21]. We found and reported the enhancement of both epithelial sodium channel (ENaC) expression and transcription along aldosterone sensitive distal nephrnon (ASDN) despite high oral salt intake by Nedd4-2 C2 KO mice. ENaCs are ion transporters that play a pivotal role in sodium transport in the terminal nephron and are representative aldosterone-inducible proteins (AIPs). The present study aimed to elucidate the effect of eplerenone (EPL) and amiloride in Nedd4-2 C2 KO mice, as well as the molecular mechanism underlying tubular-specific activation of ENaC in this genetically engineered model of salt-sensitive hypertension.

## 2. Results

### 2.1. Metabolic Data in Nedd4-2 C2^−/−^ Mice

The Nedd4-2 C2 domain is encoded by exon 2 in chromosome 18 of mice, and Nedd4-2 C2 KO mice show salt-sensitive hypertension under high oral salt intake, as reported previously [21]. There were no significant differences between the body weights of Nedd4-2 C2 KO mice and their WT (wild type) littermates, as shown in Table 1.

**Figure 1:**
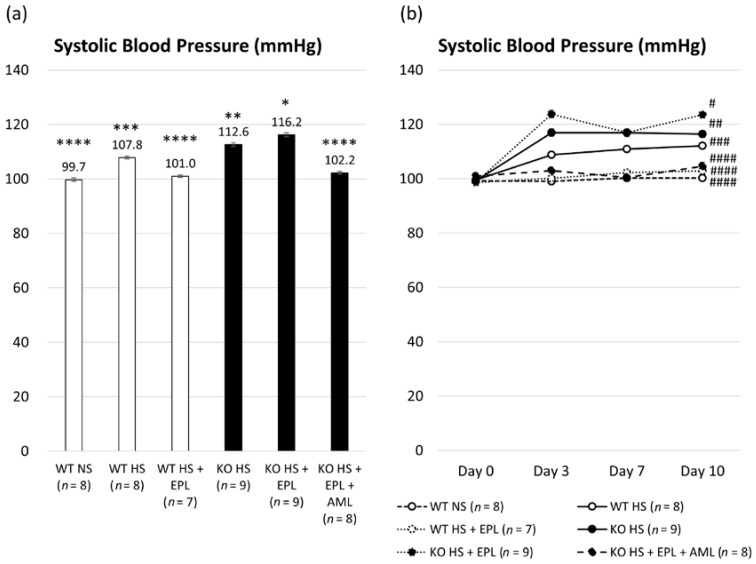
Systolic blood pressure (SBP) measured using the tail-cuff method. (**a**) Average SBP in each group for whole experimental periods analyzed by one-way ANOVA; (**b**) Average SBP on days 0, 3, 7, and 10 in each group analyzed by two-way repeated measured ANOVA. Data are from wild-type (WT) mice on a normal salt diet (NS) (*n* = 8), wild-type mice on a high-salt diet (*n* = 8), wild-type mice on a high-salt plus eplerenone (EPL) diet (*n* = 3), Nedd4-2 C2 knock out (KO) mice on a high-salt diet (*n* = 9), Nedd4-2 C2 KO mice on a high-salt (HS) plus EPL diet (*n* = 9), and Nedd4-2 C2 KO mice on a high-salt plus EPL diet, treated with amiloride (AML) (*n* = 4). * *p* < 0.01 compared with WT NS, WT HS, WT HS + EPL, KO HS, and KO HS + EPL + AML; ** *p* < 0.05 compared with WT NS, WT HS, WT HS + EPL, KO HS + EPL, and KO HS + EPL + AML; *** *p* < 0.05 compared with WT NS, WT HS + EPL, KO HS, KO HS + EPL, and KO HS + EPL + AML; **** *p* < 0.01 compared with WT HS, KO HS, and KO HS + EPL. # *p* < 0.001 compared with WT NS, WT HS, WT HS + EPL, KO HS, and KO HS + EPL + AML; ## *p* < 0.001 compared with WT NS, WT HS + EPL, KO HS + EPL, and KO HS + EPL + AML; ### *p* < 0.01 compared with WT NS, WT HS + EPL, KO HS + EPL, and KO HS + EPL + AML; #### *p* < 0.01 compared with KO HS, KO HS + EPL, and WT HS.

We measured systolic blood pressure (SBP) using the tail-cuff method. The average SBP was lower in WT littermates than that in Nedd4-2 C2 KO mice (107.8 ± 0.5 mmHg vs. 112.6 ± 0.7 mmHg, *p <* 0.001). EPL was able to reduce the SBP in WT littermates (107.8 ± 0.5 mmHg vs. 101.0 ± 0.5 mmHg, *p <* 0.001); however, no significant blood pressure reduction was observed in Nedd4-2 C2 KO mice (112.6 ± 0.7 mmHg vs. 116.2 ± 0.7 mmHg, *p <* 0.001). Thus, Nedd4-2 C2 KO mice showed EPL-resistant hypertension. Amiloride (AML) had a clear demoting effect in Nedd4-2 C2 KO mice with HS (high salt) + EPL (102.2 ± 0.5 mmHg vs. 116.2 ± 0.7 mmHg, *p <* 0.01) as shown in Figure 1a. In all groups, SBP was elevated after 3 days of a high-salt diet, as shown in Figure 1b.

**Figure 2:**
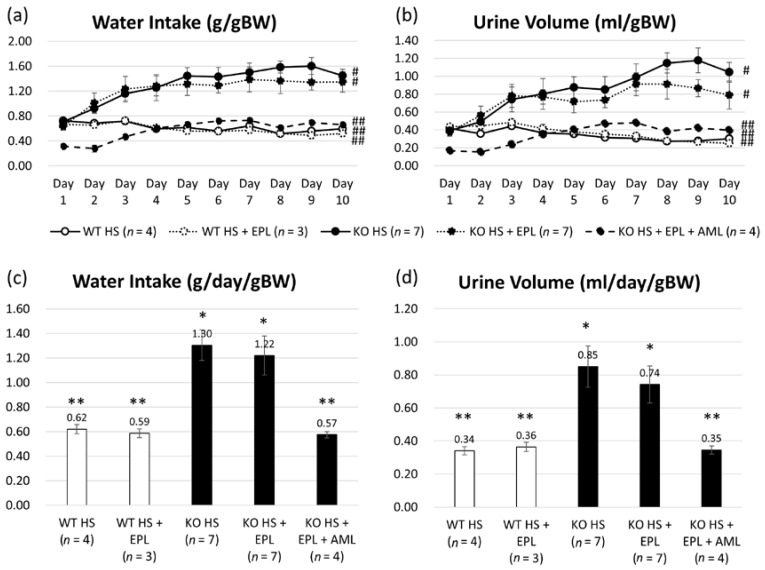
Water intake (WI) and urine volume (UV) measured daily. Averages of WI (**a**) or UV (**b**) from days 1 to 10 in each group. Analyzed by two-way repeated ANOVA. Averages of total WI (**c**) or UV (**d**) in each group analyzed by one-way ANOVA. Data are from wild-type mice on a high-salt diet (*n* = 4), wild-type mice on a high-salt plus EPL diet (*n* = 3), Nedd4-2 C2 KO mice on a high-salt diet (*n* = 9), Nedd4-2 C2 KO mice on a high-salt plus EPL diet (*n* = 9), and Nedd4-2 C2 KO mice on a high-salt plus EPL diet, treated with AML (*n* = 4). * *p* < 0.01 compared with WT HS, WT HS + EPL, and KO HS + EPL + AML; ** *p* < 0.01 compared with KO HS and KO HS + EPL. # *p* < 0.05 compared with WT HS, WT HS + EPL, and KO HS + EPL + AML; ## *p* < 0.05 compared with KO HS and KO HS + EPL.

Water intake (WI) and urine volume (UV) were measured daily. WI increased after 2 days of high-salt diet in KO mice and UV increased after 3 days in KO mice, as shown in Figure 2a,b. Based on the body weight (BW) the average WI on a high-salt diet was 0.62 ± 0.02 g/day/gBW in WT littermates and 1.30 ± 0.12 g/day/gBW in KO mice. The average UV under high-salt diet was 0.34 ± 0.02 g/day/gBW in WT littermates and 0.85 ± 0.12 g/day/gBW in KO mice as shown Figure 2c,d. For both WI and UV, there was a significant increase in KO mice compared to that in WT littermates, and there was no significant effect on WI and UV with EPL. In contrast, AML decreased both WI and UV in KO mice, similar to those in WT littermates.

**Figure 3:**
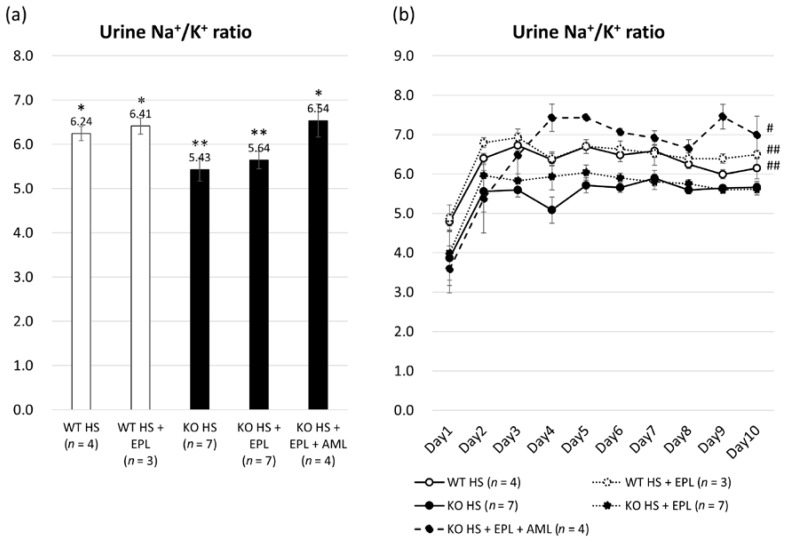
Na^+^/K^+^ ratio calculated from urine Na^+^ and K^+^ concentrations. (**a**) Averages of total Na^+^/K^+^ ratio in each group analyzed by one-way ANOVA; (**b**) Averages of Na^+^/K^+^ ratio from days 1 to 10 in each group analyzed by two-way repeated ANOVA. Data are from wild-type mice on a high-salt diet (*n* = 4), wild-type mice on a high-salt plus EPL diet (*n* = 3), Nedd4-2 C2 KO mice on a high-salt diet (*n* = 9), and Nedd4-2 C2 KO mice on a high-salt plus EPL diet (*n* = 9). * *p* < 0.01 compared with KO HS and KO HS + EPL; ** *p* < 0.01 compared with WT HS, WT HS + EPL, and KO HS + EPL + AML. # *p* < 0.05 compared with KO HS and KO HS + EPL; ## *p* < 0.05 compared with KO HS.

The urine Na^+^/K^+^ ratio was calculated daily by determining the urine sodium and potassium concentrations. A significant reduction was observed in Nedd4-2 C2 KO mice compared to WT littermates (5.06 ± 0.20 vs. 6.25 ± 0.16, *p <* 0.001), and EPL generally increased the Na^+^/K^+^ ratio both in Nedd4-2 C2 KO mice and WT littermates (5.50 ± 0.16 and 6.41 ± 0.18), as shown in Figure 3a,b. This indicates that sodium reabsorption via ENaCs was increased in Nedd4-2 C2 KO mice and that EPL inhibited the function of renal outer medullary K+ channel (ROMK). AML inhibited ENaCs, and the Na^+^/K^+^ ratio increased in Nedd4-2 C2 KO mice similar to that in WT littermates.

### 2.2. Quantitative Analysis of Epithelial Sodium Channel (ENaC) Transcripts

**Figure 4:**
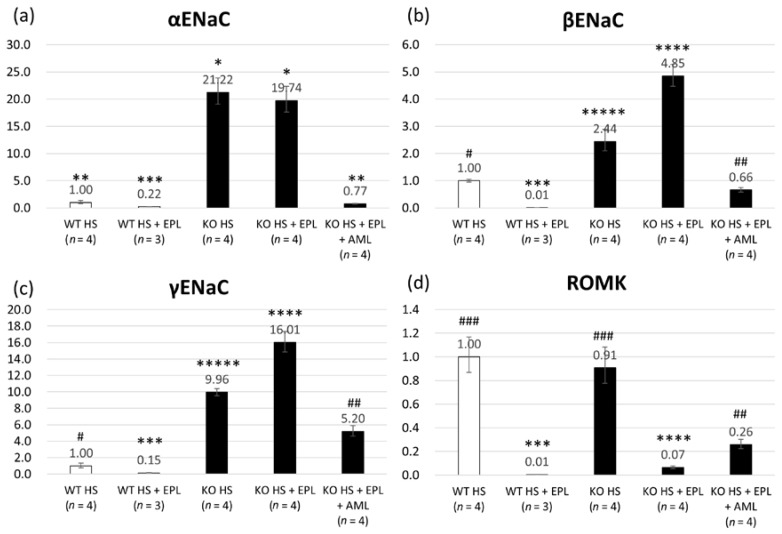
Total RNA abundance of (**a**) α *ENaC*; (**b**) β *ENaC*; (**c**) γ *ENaC*; and (**d**) *ROMK* in laser-captured distal tubules/connecting tubules/cortical collecting ducts analyzed by one-way ANOVA. Data are from wild-type mice on a high-salt diet (*n* = 4), wild-type mice on a high-salt plus EPL diet (*n* = 3), Nedd4-2 C2 KO mice on a high-salt diet (*n* = 4), and Nedd4-2 C2 KO mice on a high-salt plus EPL diet (*n* = 4). * *p* < 0.01 compared with WT HS, WT HS + EPL, and KO HS + EPL + AML; ** *p* < 0.01 compared with WT HS + EPL, KO HS, and KO HS + EPL; *** *p* < 0.01 compared with WT HS, KO HS, KO HS + EPL, and KO HS + EPL + AML; **** *p* < 0.01 compared with WT HS, WT HS + EPL, KO HS, and KO HS + EPL + AML; ***** *p* < 0.01 compared with WT HS, WT HS + EPL, KO HS + EPL, and KO HS + EPL + AML. # *p* < 0.01 compared with WT HS + EPL, KO HS, KO HS + EPL, and KO HS + EPL + AML; ## *p* < 0.01 compared with WT HS, WT HS + EPL, KO HS, and KO HS + EPL; ### *p* < 0.01 compared with WT HS + EPL, KO HS + EPL, and KO HS + EPL + AML.

The expression of α, γ, and β *ENaC* in ASDN was measured by quantitative RT-PCR analysis of microdissected tissue samples. Compared with those in WT littermates, *ENaC* transcripts were increased significantly in Nedd4-2 C2 KO mice, and EPL did not suppress their expression in Nedd4-2 C2 KO mice, as shown in Figure 4a–c. These results demonstrate that the activation of *ENaC* transcripts in ASDN occurs independently of aldosterone-mineral corticoid receptor (MR) signaling.

### 2.3. Semi-Quantitative Analysis of ENaC Proteins

**Figure 5:**
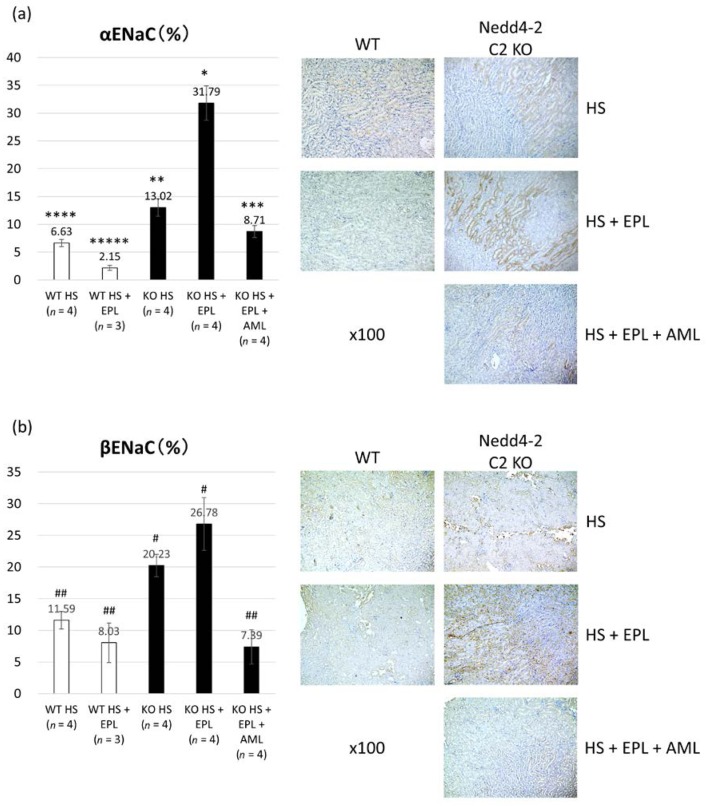

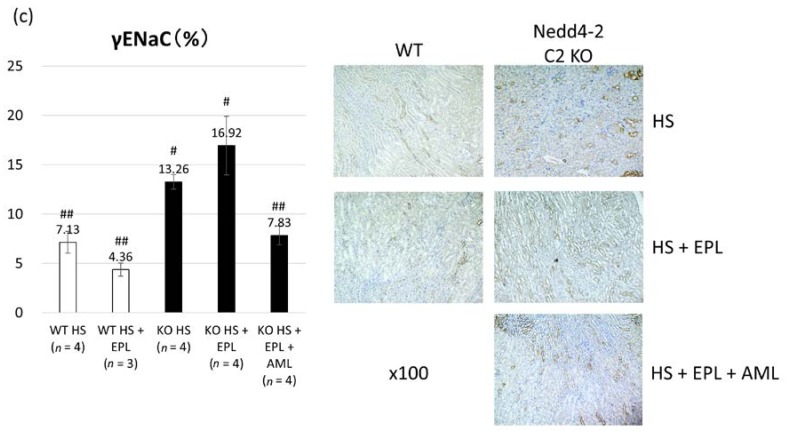
Immunostaining of (**a**) α ENaC; (**b**) β ENaC; and (**c**) γ ENaC proteins by immunohistochemistry. Data are from wild-type mice on a high-salt diet (*n* = 4), wild-type mice on a high-salt plus EPL diet (*n* = 3), Nedd4-2 C2 KO mice on a high-salt diet (*n* = 4), and Nedd4-2 C2 KO mice on a high-salt plus EPL diet (*n* = 4). * *p* < 0.01 compared with WT HS, WT HS + EPL, KO HS, and KO HS + EPL + AML; ** *p* < 0.05 compared with WT HS, WT HS + EPL, and KO HS + EPL; *** *p* < 0.05 compared with WT HS + EPL and KO HS + EPL; **** *p* < 0.05 compared with WT HS + EPL, KO HS, and KO HS + EPL; ***** *p* < 0.05 compared with WT HS, KO HS, KO HS + EPL, and KO HS + EPL + AML. # *p* < 0.01 compared with WT HS, WT HS + EPL, and KO HS + EPL + AML; ## *p* < 0.01 compared KO HS and KO HS + EPL.

We conducted staining to detect α, β, or γ ENaC expression in the kidneys by using specific antibodies and then determined the ratios of the stained area using the cell-count method. Semi-quantitative histopathological examination of ENaCs revealed significant differences between the four groups, as shown in Figure 5a–c. Similar to the transcript analysis, there was significant elevation in the expression of each ENaC in KO mice, and EPL did not inhibit ENaC activation. Therefore, ENaC proteins were enhanced in KO mice and independent of aldosterone in ASDN.

## 3. Discussion

ENaCs are ion transporters expressed along the ASDN that play a pivotal role in handling sodium in the terminal nephron. ENaC activity in the terminal nephron is regulated by open probability, expression levels (regulation of expression), and membrane abundance (post-translational modification). Genetic analyses of representative familial hereditary hypertension, known as Liddle syndrome, showed that the Proline-Tyrosine (PY) motif in the C terminus of ENaCs are commonly mutated, resulting in impaired protein-protein interactions with the Nedd4L Tryptophan-Tryptophan (WW) domain [22]. Failure of post-transcriptional modifications (ubiquitination) and subsequent trafficking to the proteasome result in persistent enhancement of membranous ENaC abundance and salt-sensitive hypertension. The total amount of sodium reabsorption in the terminal nephron depends on the ENaC expression level in the membrane, and accelerated reabsorption of intra-tubular sodium results in the development of salt-sensitive hypertension in vivo. Our detailed genetic analyses revealed the molecular diversity of the *Nedd4-2/L* gene in both humans and rodents, and two to three isoforms with and without the C2 domain in their N-terminus [23,24]. Subsequent analyses with the originally developed polyclonal antibodies for the C2 domain revealed specific expression in ASDN and colocalization of Nedd4-2/L with the C2 domain and ENaCs in the terminal nephron [24,25]. Therefore, we hypothesized that the Nedd4-2 C2 isoform functions as a critical ubiquitination enzyme involved in ENaC degradation in the ASDN.

Isoform-specific gene targeting strategies showed that Nedd4-2 C2 KO mice are salt-sensitive and impair post-transcriptional modification of the ENaC proteins [21]. Our previous analyses demonstrated linearly enhanced expression of tubular ENaCs in accordance with genotype and oral salt intake, which is abolished by amiloride treatment. These findings suggest that the amplified intracellular sodium signaling resulted in *ENaC* gene transcription, which is known as excitation and transcription coupling of monovalent cations [26,27,28,29]. ENaC is considered a major effector of aldosterone-mediated sodium reabsorption and fluid regulation. Therefore, ENaCs are representative aldosterone-inducible proteins (AIP). Here, we demonstrated that the originally developed Nedd4-2 C2 KO mice show salt-sensitive hypertension, which was EPL-resistant (Figure 1). Detailed molecular and histopathological analyses of the ASDN demonstrated that EPL treatment failed to suppress both ENaC mRNA and protein expression in KO mice, whereas significant suppression of both was observed in wild-type littermates (Figure 4 and Figure 5). Analyses of the urine Na^+^/K^+^ ratios and quantitative RT-PCR for laser-captured ASDN showed that ROMKs, which are also representative AIPs and function in potassium secretion from ASDN, are suppressed by EPL treatment as expected (Figure 3 and Figure 4). Thus, we found that the action of EPL on the ASDN in Nedd4-2 C2 KO mice deviated between ENaC and ROMK for electrolyte reabsorption and secretion. This may be tested endocrinologically by measuring the aldosterone/corticosterone ratio, which is one of the limitations of our current experiments. However we found no significant pathological change in the glomerulus by light microscopic examinations, and thus urine albumin/protein secretion may be necessary to address kidney functions for our current model of salt-sensitive hypertension with pharmacological treatment.

Aldosterone-independent activation of ENaC occurred in part due to the activation of the tubular renin-angiotensin system. We previously demonstrated that a high-sodium diet upregulated ENaC expression despite decreased plasma aldosterone concentrations [30]. Aldosterone-independent upregulation of ENaC expression was also observed in Dahl salt-sensitive rats by Kakizoe et al., which was caused by the post-transcriptional cleavage of the inactivation domains of γ ENaC [31] by channel-activating enzymes such as prostacin and furin [32,33,34]. Additionally, Nagase et al. reported that Rac1, a rho family small GTP-binding protein, accelerated mineral corticoid receptor transactivation independent of aldosterone [35]. These are considered among the various molecular mechanisms involved in the paradoxical activation of ENaC under high-oral salt intake resulting in salt sensitivity and hypertension. In this study, enhanced ENaC expression was observed despite EPL treatment, whereas amiloride treatment successfully reduced blood pressure elevation and *ENaC* gene expression in Nedd4-2 C2 KO mice with high oral salt intake and EPL treatment (Figure 1, Figure 4 and Figure 5). Moreover, detailed experiments using metabolic cages and daily urine collection revealed that both water intake and urine volume abnormalities were fully restored to the normal state by amiloride treatment (Figure 2). Amiloride treatment may also suppress sodium reabsorption through ENaC itself and subsequent excitation-transcription coupling because of increased intracellular sodium, which is independent of aldosterone-MR signaling. These results suggest that enhanced sodium reabsorption through ENaC accelerated *ENaC* gene expression under loss of the specific Nedd4-2 isoform. Thus, the artificially genetic-engineered model of Nedd4-2 C2 loss revealed the underlying mechanism of salt sensitivity, which may account for resistant hypertension. Further studies are needed to examine the relationships between intracellular Na and *ENaC* gene expression (known as excitation and transcription coupling of monovalent cations).

Interestingly, Melander et al. reported a subject with rs4149601, a common variant of human Nedd4L formed by activating a cryptic splice site resulting in a frameshifted transcript [23], which showed low renin salt-sensitive hypertension with significantly high cardiovascular mortalities in a prospective clinical observational study [36,37]. Our in vitro analyses using *Xenopus* oocyte heterologous expression systems revealed that subjects with the rs4149601 A to G mutation showed impaired ENaC ubiquitination by the dominant-negative effects of specific Nedd4L isoforms [25]. However, whether subjects with rs4149601 A to G showed EPL resistance remains unclear. Our current analyses showed that amiloride or similar pharmaceutical products such as triamterene are potent effective options for treating resistant hypertension and salt sensitivity in order to correct and optimize the tubular mechanism underlying the disease.

## 4. Materials and Methods

### 4.1. Metabolic Studies

Age-matched (15.2 ± 0.4 weeks old) male Nedd4-2 C2^−/−^ mice (KO, *n* = 22) with a C57Bl6/J background and their wild-type littermates (WT, *n* = 7) were kept for 10 days in individual metabolic cages (SN-781, Shinano Manufacturing Co., Ltd., Tokyo, Japan) under controlled light, temperature, and humidity after a 4-day habituation period. They were divided into five groups and received the following diets: (1) WT mice were given a high-salt diet containing 8% NaCl (WT HS group, *n* = 4); (2) WT mice were given a high-salt diet containing 8% NaCl plus eplerenone (WT HS + EPL group, *n* = 3); (3) Nedd4-2 C2 KO mice were given a high-salt diet containing 8% NaCl (Nedd4-2 C2 KO HS group, *n* = 9); (4) Nedd4-2 C2 KO mice were given a high-salt diet containing 8% NaCl plus eplerenone (Nedd4-2 C2 KO HS + EPL group, *n* = 9); and (5) Nedd4-2 C2 KO mice were given a high-salt diet containing 8% NaCl plus eplerenone and amiloride (Nedd4-2 C2 KO HS + EPL + AML group, *n* = 4). Eplerenone (Pfizer, Inc., New York, NY, USA) was mixed into the diets at a dose of 1.25 g/kg of chew). Amiloride (Biomol International, LP, Plymouth Meeting, PA, USA) was administered daily by intra-abdominal injection at a dose of 1 mg/kg/day. Body weight (BW), water intake volume, and urine volume were measured, and daily urine samples were collected. The concentrations of urine sodium and potassium were measured using specific electrodes (Oriental Yeast Co., Ltd., Tokyo, Japan). Systolic blood pressure (SBP) and heart rate (HR) were recorded with the tail-cuff method (MK-2000, Muromachi Kikai Co., Ltd., Tokyo, Japan) on days 0, 3, 7, and 10. On day 11, the mice were sacrificed under anesthesia with sevoflurane, and their kidneys were dissected and fixed for subsequent total RNA extraction and immunohistopathological analyses. Blood pressure measurements were performed by two independent observers, who were blinded to the details of the experiments. All animal experiments were performed in accordance with the guidelines of the Animal Experiment Committee, Yokohama City University School of Medicine, and with approval of the Animal Experiment Committee, Yokohama City University School of Medicine (F-A-14-088).

### 4.2. Quantitative Reverese Transcriptase Polymerase Chain Reaction (RT-PCR) Analyses of ENaC Transcripts

Total RNA abundance of α, β and γ ENaCs in laser-microdissected late distal convoluted tubules/connecting tubules/cortical collecting ducts (DCT/CNT/CCD) were measured by quantitative RT-PCR analysis. We used TaqMan probes for mouse α ENaC (Mm00803386_m1), β ENaC (Mm00441215_m1), and γ ENaC (Mm00441228_m1), all of which were purchased from Applied Biosystems (Foster City, CA, USA). Kidney sections were stained with an anti-calbindin D28K antibody, which was used as a marker of late DCT/CNT/CCD. Specific regions, expressing calbindin D-28K, were carefully microdissected using a Leica CTR 6000 (Leica Microsystems, Wetzlar, Germany) and collected into the cap of a 0.2-mL microtube. The total area of microdissection was 5,000,000 µm^2^ in each group. The tissues were then pooled for total RNA extraction using an RNeasy FFPE Kit (Qiagen, Hilden, Germany). Synthesis of cDNA from total RNA was carried out using High-Capacity RNA-to-cDNA Master Mix (Applied Biosystems). All PCRs were performed three times in duplicate.

### 4.3. Semi-Quantitative Immunohistopathological Analysis of ENaC Proteins

The area of ENaCs stained with anti-α, β, and γ ENaC antibodies (kindly provided by Dr. Kitamura and Dr. Kakizoe, Yamanashi University, Kofu, Japan, Kumamoto University, Kumamoto, Japan) in each DCT/CNT/CCD tubule selected was calculated using a computerized touch pen device under a BZ-9000 microscope with Dynamic Cell Count BZ-HIC image analysis software (Keyence, Osaka, Japan). These antibodies were originally developed using a specific epitope common to those of Masilamani, et al. Specifically, the αENaC antibody was raised against the N-terminal peptide (amino acids 46–68; NH_2_-LGKGDKREEQGLGPEPSAPRQPTC-COOH), while the βENaC and γENaC antibodies were raised against the C-terminal peptides (βENaC: amino acids 617–638; NH_2_-CNYDSLRLQPLDTMESDSEVEAI-COOH, γENaC: amino acids 629–650; NH_2_-CNTLRLDRAFSSQLTDTQLTNEL-COOH) according to Masilamani, et al. [38]

### 4.4. Statistical Analyses

Data are expressed as the mean ± standard error (SE). All statistical analyses were performed with SPSS version 21.0 software (SPSS, Inc., Chicago, IL, USA) and R 3.2.2 (R Foundation for Statistical Computing, Vienna, Austria. Available online: https://www.R-project.org/). The relative abundance of mouse transcripts and proteins in the kidney were compared statistically by one-way analysis of variance (ANOVA) followed by Bonferroni’s multiple comparison tests. *p* < 0.05 was considered to indicate statistical significance.

## 5. Conclusions

We found Nedd4-2 C2 KO mice show EPL resistant salt-sensitive hypertension and amiloride treatment restore abberant enhancement of *ENaC* gene transcription along ASDN. This suggested enhanced sodium reabsorption through ENaC itself accelerated *ENaC* gene expression under loss of the specific Nedd4-2 isoform, which may account for one explanatory mechanism for resistant hypertension.

## Figures and Tables

**Table 1:** Characteristics of body weight (BW) between day 0 and day 10.

	WT Littermates			Nedd4-2 C2^−/−^ (KO)			*p* ^1^
	NS (*n* = 8)	HS (*n* = 8)	HS + EPL (*n* = 7)	HS (*n* = 9)	HS + EPL (*n* = 9)	HS + EPL + AML (*n* = 8)	
BW on day 0	28.50 ± 0.53	27.42 ± 1.17	26.78 ± 0.98	26.75 ± 0.93	27.69 ± 1.09	29.76 ± 0.68	0.2133
BW on day10	29.43 ± 0.53	28.02 ± 1.09	26.69 ± 1.30	27.86 ± 0.60	26.80 ± 1.06	28.59 ± 0.40	0.2584

^1^ Analyzed by one-way analysis of variance (ANOVA).

## Abbreviations

ASDN	Aldosterone Sensitive Distal Nephron
ENaC	Epithelial Sodium Channel
EPL	Eplerenone
